# Abdominal Wall Necrotizing Fasciitis in a Preemie: A Case Report of a Fulminant Course

**DOI:** 10.1155/crdi/5555074

**Published:** 2026-05-06

**Authors:** Hari Narayan Rai, Sagar Kafle, Smriti Basnet, Prabineshwor Prasad Lekhak, Aakash Mishra, Saukat Ali, Sunil Raja Manandhar, Ashish Lal Shrestha

**Affiliations:** ^1^ Department of Pediatric and Neonatal Surgery, Kathmandu Medical College Teaching Hospital, Kathmandu, Nepal, kmc.edu.np; ^2^ Department of Neonatology, Kathmandu Medical College Teaching Hospital, Kathmandu, Nepal, kmc.edu.np

**Keywords:** abdominal wall, *Klebsiella*, necrotizing fasciitis, neonatal sepsis, neonatal surgery, preterm

## Abstract

Necrotizing fasciitis (NF), a rapidly progressive soft‐tissue infection characterized by extensive tissue necrosis, is a fulminant condition that, if not treated promptly, can become fatal. Predominantly a disease of adulthood, its occurrence in the pediatric and neonatal populations is rare. We report a case of a preterm newborn who developed NF of the abdominal wall due to the rare etiology of invasive *Klebsiella pneumoniae* in the setting of culture‐proven sepsis secondary to ventilator‐associated pneumonia. This case highlights the challenges of early recognition and management in unstable preterm neonates with rapidly progressive infections, in whom a fatal outcome could not be avoided despite timely and intensive management efforts.

## 1. Introduction

Necrotizing fasciitis (NF), a rare, aggressive and often a life‐threatening infection, stands out with a global incidence of 0.3–15.5 cases/100,000 population and a mortality rate of almost 20% [1–3]. The infective process frequently involves a polymicrobial etiology that can spread through all the layers of skin often involving subcutaneous tissue, fascia, and even extending up to the muscular layer. In newborns, NF has a mortality rate of 24.1% and often a history of previous trauma.

An infective association may be noted in up to 80% that may include conditions like balanitis, omphalitis, or necrotizing enterocolitis. Sometimes, factors as trivial as vaccination, venous access line, scalp or chest electrodes, or even unhealthy birth practices may be a causative attribute to the disease [1, 4]. We hereby present a preterm neonate with a fulminant course of NF complicated by ventilator‐associated pneumonia (VAP) associated with *Klebsiella* sepsis.

## 2. Case Report

A baby girl born at 32 weeks and 4 days of gestational age (birth weight: 1680 g) following a vaginal delivery to nonconsanguineous parents was soon transferred to the neonatal intensive unit and intubated in view of respiratory distress. With a working diagnosis of hyaline membrane disease, surfactant therapy was initiated using the ENSURE method, simultaneously sending blood culture and administering intravenous (IV) antibiotics.

**FIGURE 1:**
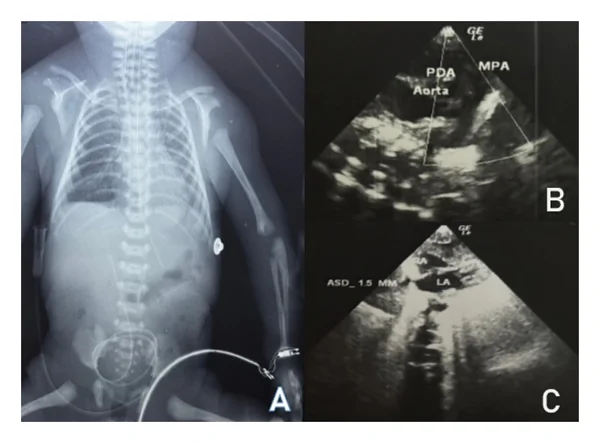
(A) Chest and abdomen x‐ray in AP view; (B) echocardiography finding showing patent ductus arteriosus; and (C) echocardiography finding showing atrial septal defect.

At 48 h of life, a gradual clinical deterioration was observed with swinging pyrexia, tachycardia, and bilateral chest rhonchi coupled with frequent episodes of oxygen desaturations requiring escalations in ventilatory support. An instantaneous hemogram showed leucopenia. A diagnosis of VAP was made, and IV antibiotics were upgraded. Also, a bedside echocardiography revealed an atrial septal defect and a patent ductus arteriosus each measuring 1.5 mm (Figure 1).

A follow‐up hemogram at 72 h showed neutropenia on a preexisting leukopenia while C‐reactive protein also turned positive. This was soon followed by the development of hemodynamic instability requiring inotropic support. Septic shock was diagnosed in the background of late‐onset neonatal sepsis, and conservative management was continued. Meanwhile, the blood culture sent at admission was reported to be sterile.

Day 4 of life marked the beginning of acute kidney injury (AKI) with gradual onset of oliguria and simultaneous elevation in serum creatinine levels (1.6 mg/dL). In view of clinical worsening, IV antibiotics were upgraded to colistin in a renal adjusted dosage after a second blood culture while continuing intensive monitoring and supportive care. Of particular clinical note was diffuse and generalized skin hardening that seemed to spare only her palms, soles, and genitalia. This was attributed to sclerema neonatorum, for which IV immunoglobulin therapy was instituted.

**FIGURE 2:**
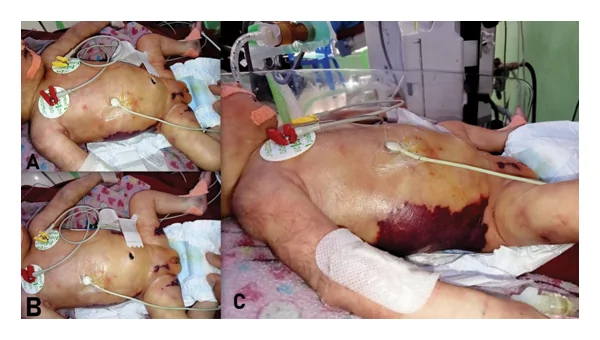
(A) Lesions over the bilateral anterolateral abdominal wall; (B) lesions over the umbilicus, groin and medial aspect of bilateral thighs; and (C) lesion over the right flank.

On the 8th day of life, sharply defined reddish‐purple erythematous lesions with swelling and edema (5.2 × 2.5 cm in the largest dimension) were noted that were characteristically more over the lower abdomen and flanks (Figure 2). While the hemogram showed persistent leukopenia (white blood count of 1700 cells/mm^3^) with increasing CRP levels (266.9 mg/L: reference normal level < 10 mg/L), an abnormal coagulation profile (prothrombin time: 28 s, control: 14 s, and international normalized ratio: 2.0) was also seen in addition to persisting renal dysfunction (serum urea: 94 mg/dL and serum creatinine: 1.3 mg/dL).

A Laboratory Risk Indicator for Necrotizing Fasciitis (LRINEC) Score of 7 was calculated based on the clinical appearance, background of sepsis, and laboratory parameters, indicating a 50%–75% probability of NF. Meanwhile, the second blood culture sample reported growth of *Klebsiella pneumoniae* sensitive to IV colistin that she was already receiving. Considering her critical clinical condition and inability to tolerate surgical stress, a conservative line of management was offered rather than radical debridement along with close observation and ongoing support.

Despite the best medical care, disease progression was seen extending up to the umbilicus, groin, and medial aspect of bilateral thighs. She eventually developed metabolic acidosis with florid coagulopathy that resulted in pulmonary hemorrhage and bleeding from the venous access sites.

A rapidly worsening condition then ensued, resulting in hypotension, persistent bradycardia, and repeated episodes of cardiac asystole. On the 10th day of life, she was declared to be deceased.

## 3. Discussion

NF is an aggressive infection of the skin, soft tissue, and muscle fascia that starts with nonspecific findings like swelling and erythema of the skin, later progressing to cause induration, sclerosis, putrid discharge, bullae formation, and eventually leading to extensive soft tissue necrosis, systemic shock, and multiorgan dysfunction, frequently culminating in fatality [3, 4].

With a global prevalence of 0.4 cases/100,000 population, NF usually affects immunocompromised adults. However, in the pediatric population, this occurrence accounts for 0.08–1 case/100,000 children worldwide, thereby continuing to be a condition that is poorly studied or understood, and even more so amongst the neonates [3].

Although mostly a clinical diagnosis, scoring systems based on clinical and laboratory parameters can aid in the diagnosis of NF. A score: LRINEC was originally developed for a cohort of adult patients and later modified for children, as P‐LRINEC seems to demonstrate a greater accuracy in predicting pediatric necrotizing soft tissue infection [5]. This score incorporates serum CRP and sodium levels, with a CRP value > 20 mg/L making it most sensitive and a serum sodium level < 135 mg/dL making it most specific in the diagnosis of pediatric NF [5]. Although the P‐LRINEC score appears to offer improved predictive accuracy for necrotizing soft tissue infections in pediatric populations, it has been validated only in older children. Its applicability to neonates, particularly preterm infants, has not been established. In preterm infants, clinical judgment should take precedence over scoring systems that have not been validated in this population.

In a comprehensive review of 76 reported cases of neonatal NF, *Klebsiella* species were identified as the causative pathogen in only three cases, involving the perianal region in one neonate, the genital region with lower abdominal extension in another, and the scalp in a third [1]. In most of the reported cases, the back of the torso was found to be the frequent site of involvement, followed by the abdomen and chest. While some authors reported a possible inducing event like omphalitis and bullous impetigo or more severe systemic infections and immunodeficiency, even procedures as trivial as circumcision or electrodes for the monitoring of vital signs were identified as a trigger in a few [1, 6, 7]. In the index case, however, the site of involvement was the abdominal wall, and possibly VAP could have triggered the event.

The most recent series from Iran mentions a mean age of 13.06 ± 8.66 days, with 55.7% of the cases in term and only 17.7% in preemies along with a polymicrobial cause (63.29% Gram‐positive and 21.5% Gram‐negative) [1]. Among Gram‐positives, *Staphylococcus* and *Streptococcus* were most frequently identified [1, 7]. However, in the index case, *Klebsiella pneumoniae*, a Gram‐negative bacterium, was isolated in the blood that was suspected of inciting the event.

The initial diagnosis of sclerema neonatorum on Day 4 was based on diffuse, uniform skin induration in a critically ill premature neonate with septic shock, consistent with its typical clinical presentation. Early abdominal ultrasound was inconclusive, lacking hallmark features of NF, which may be absent in the early stages of disease. The initial blood culture was negative, while a second sample sent on Day 4 subsequently grew *Klebsiella pneumoniae* sensitive to IV colistin, which the patient was already receiving. The subsequent development of sharply demarcated violaceous erythematous lesions with edema on Day 8 supported the diagnosis of NF, highlighting the diagnostic challenge and potential for delayed recognition in unstable preterm neonates.

While optimum coverage with broad‐spectrum antibiotics and vigilant adjustment based on culture reports is imperative, appropriate local wound care is paramount. Of the available options, honey seems to be the favored form of dressing in most of the successfully treated cases of newborn NF [1].

Conventional management of NF includes aggressive surgical debridement in addition to broad‐spectrum antimicrobial therapy and supportive care, with adjunctive measures such as immunomodulation and hyperbaric oxygen therapy used infrequently. Surgical debridement remains the primary intervention and is recommended on an emergent basis, as delays are associated with increased mortality, and current guidelines do not specify absolute contraindications to surgery. However, with the index patient having low birth weight, supposedly low immune response, and a gamut of events like septic shock, renal failure, and multiorgan dysfunction on top of prematurity, associated hyaline membrane disease, and cardiac findings, we could not offer an aggressive surgical plan. Surgery was, therefore, deferred following multidisciplinary assessment that the infant was unlikely to survive the immediate perioperative period. This decision was informed by extreme prematurity with septic shock, florid coagulopathy posing a high risk of uncontrollable hemorrhage, multiorgan failure requiring escalating cardiorespiratory support, and extensive disease necessitating radical and potentially repeated debridement. Thus, the decision to defer surgery was based on concerns regarding perioperative feasibility and anticipated mortality risk. Despite maximal conservative and supportive medical management, the newborn could not be saved.

Owing to the fatal outcome, a clinical autopsy or detailed postmortem examination could have yielded valuable diagnostic and educational insights; however, parental refusal precluded this assessment, constituting a limitation of the study.

## 4. Conclusion

NF in preterm neonates is rare but can present with a rapidly fulminant and deteriorating course, often with adverse outcomes. Clinical vigilance, early recognition, and prompt treatment remain the cornerstones of management.

## Author Contributions

All authors attest that they meet the current ICMJE criteria for authorship.

## Funding

This research did not receive any funding.

## Ethics Statement

This case report is exempt from ethical approval due to the nature of the article, as per the ethical review board at our institution.

## Consent

Informed consent was obtained from the guardian of the patient.

## Conflicts of Interest

The authors declare no conflicts of interest.

## Data Availability

The data that support the findings of this study are available from the corresponding author upon reasonable request.
